# Do integrated health and social care services deliver better care for children with intellectual disabilities?

**Published:** 2012-09-04

**Authors:** Axel Kaehne

**Affiliations:** Cardiff University, School of Medicine, UK

**Keywords:** intellectual disabilities, mental retardation, integration, health, social care, partnerships, evaluation

## Abstract

**Purpose:**

The project focussed on two local services in Wales that have recently moved to (what they call) an integrated health and social care intellectual disabilities service.

**Research questions:**

What are the advantages and disadvantages of service integration as viewed by parents with respect to care provision for young people with intellectual disabilities?

**Method:**

We conducted a parental survey facilitated by nursing staff in both local services.

**Results:**

Parents were generally skeptical of any positive impact of organisational changes on quality of care for children with LD. The main issues of concern for parents, however, did NOT relate to integration but to lack of resources and quality of care provision. This has serious implications for the way in which we should evaluate organisational changes such as health and social care integration for this user group.

**Conclusion:**

Parental concerns cover a wide range of issues. These issues reflect the unique needs of service users. Evaluating the impact of service integration for service users need to take account of different needs and abilities in this population group. The study also helps us to appreciate the limitations of parental surveys to measure service integration outcomes.

Powerpoint presentation available at http://www.integratedcare.org at congresses – San Marino – programme.

